# Neurodevelopmental Expression Profile of Dimeric and Monomeric Group 1 mGluRs: Relevance to Schizophrenia Pathogenesis and Treatment

**DOI:** 10.1038/srep34391

**Published:** 2016-10-10

**Authors:** Jeremy S. Lum, Francesca Fernandez, Natalie Matosin, Jessica L. Andrews, Xu-Feng Huang, Lezanne Ooi, Kelly A. Newell

**Affiliations:** 1 Illawarra Health and Medical Research Institute, Wollongong, New South Wales 2522 Australia; 2 School of Medicine, Faculty of Science, Medicine and Health, University of Wollongong, Wollongong, NSW 2522 Australia; 3 Schizophrenia Research Institute, Sydney, NSW 2010 Australia; 4 School of Psychology, Faculty of Science, Medicine and Health, University of Wollongong, Wollongong, NSW 2522, Australia; 5 Department of Translational Research in Psychiatry, Max Planck Institute of Psychiatry, Kraepelinstrasse 2-10 Munich Germany; 6 School of Biological Sciences, Faculty of Science, Medicine and Health, University of Wollongong, Wollongong, NSW 2522 Australia

## Abstract

Group 1 metabotropic glutamate receptors (mGluR1/mGluR5) play an integral role in neurodevelopment and are implicated in psychiatric disorders, such as schizophrenia. mGluR1 and mGluR5 are expressed as homodimers, which is important for their functionality and pharmacology. We examined the protein expression of dimeric and monomeric mGluR1α and mGluR5 in the prefrontal cortex (PFC) and hippocampus throughout development (juvenile/adolescence/adulthood) and in the perinatal phencyclidine (PCP) model of schizophrenia. Under control conditions, mGluR1α dimer expression increased between juvenile and adolescence (209–328%), while monomeric levels remained consistent. Dimeric mGluR5 was steadily expressed across all time points; monomeric mGluR5 was present in juveniles, dramatically declining at adolescence and adulthood (−97–99%). The mGluR regulators, Homer 1b/c and Norbin, significantly increased with age in the PFC and hippocampus. Perinatal PCP treatment significantly increased juvenile dimeric mGluR5 levels in the PFC and hippocampus (37–50%) but decreased hippocampal mGluR1α (−50–56%). Perinatal PCP treatment also reduced mGluR1α dimer levels in the PFC at adulthood (−31%). These results suggest that Group 1 mGluRs have distinct dimeric and monomeric neurodevelopmental patterns, which may impact their pharmacological profiles at specific ages. Perinatal PCP treatment disrupted the early expression of Group 1 mGluRs which may underlie neurodevelopmental alterations observed in this model.

Glutamate is the primary excitatory neurotransmitter in the central nervous system. It plays a vital role in neurodevelopmental processes, including synaptic plasticity, neurite outgrowth and neuronal migration^1^,^2^. Glutamate signals through the ionotropic (N-methyl-D-aspartate (NMDA), α-amino-3-hydroxy-5-methyl-4-isoxazolepropionic acid (AMPA) and kainate) and metabotropic (mGluR 1–8) families of glutamate receptors. mGluRs are categorised into 3 groups, based on their sequence homology, G-protein coupling and pharmacology^3^. Group 1 mGluRs, which consist of mGluR1 and mGluR5, are expressed in the brain from early development and are implicated in the pathogenesis and treatment of several neurodevelopmental and psychiatric disorders, including schizophrenia (for review see ref. 4). mGluR1 is commonly found as 2 isoforms (mGluR1α, mGluR1β), which differ in their C-terminal and display different signalling kinetics, with the shorter variant, mGluR1β, displaying slower Ca^2+^ kinetics than mGluR1α^5^,^6^. mGluR5 is most commonly spliced as 2 variants (mGluR5a and mGluR5b), which differ by a 33 amino acid insert at the C-terminal and are neurodevelopmentally regulated^7^, yet show no pharmacological differences^8^.

The neurodevelopmental protein expression of Group 1 mGluRs has previously been reported in rodents^5^,^6^,^7^. However, many of these studies were performed before the discovery of mGluR homodimerisation and focused only on the monomeric form of these receptors. Evidence has shown that Group 1 mGluR homodimers are joined via a disulphide bond at the Venus fly trap domain located on the N-terminus^9^. Accordingly, it has been repeatedly reported that under non-reducing electrophoresis conditions, Group 1 mGluRs primarily migrate at twice the expected molecular weight^7^,^9^,^10^, consistent with their expression as constitutive homodimers. While, there is *in vitro* evidence of functional mGluR1/5 heterodimers^11^, there is still a lack of evidence for this *in vivo*. Furthermore, the functional and pharmacological consequences of Group I mGluR heteromeric complexes are still not understood. Recently, El Moustaine and colleagues^12^, highlighted the functional significance of mGluR homodimerisation, demonstrating that although dimerisation of mGluRs is not required for G-protein coupling, dimerisation is necessary for agonist-induced activation, rendering the monomeric form inactive to agonist-induced activation. Furthermore, positive allosteric modulator (PAM) compounds, which are being investigated as a novel schizophrenia treatment, act as full agonists on mGluR monomers in the absence of glutamate, with dimeric arrangement of mGluRs vital for controlling PAM-induced agonist-like activity.

Administration of NMDA receptor (NMDAR) antagonists, such as phencyclidine (PCP), during early post-natal development in rodents is widely used to investigate the neurodevelopmental aspects of schizophrenia^13^. We and others have shown that this model alters development of the glutamatergic system including the ionotropic NMDA receptor^14^,^15^,^16^,^17^. However, it is unclear whether perinatal NMDA receptor antagonism, impacts the neurodevelopmental expression of Group 1 mGluRs. These receptors are purported to be involved in the development of schizophrenia^18^,^19^,^20^,^21^,^22^ and we have recently reported that the expression of these receptors are altered in the prefrontal cortex (PFC) and hippocampus in postmortem tissue from schizophrenia subjects^23^,^24^,^25^. Understanding the developmental expression profile of Group 1 mGluRs may provide insight into their role in the development of neuropsychiatric disorders at critical neurodevelopmental time points. Therefore, the present study aimed to measure the native dimeric and monomeric forms of mGluR1α and mGluR5 throughout normal neurodevelopment and following perinatal PCP treatment, an established rodent model of schizophrenia. In addition, we examined two key regulators of Group I mGluRs, Homer1b/c and Norbin (Neurochondrin-1)^26^. To our knowledge, this is the first measure of Homer1b/c and Norbin throughout neurodevelopment in rats. Homer1b/c and Norbin increase Group 1 mGluR cell-surface expression and signalling, including the capacity of mGluR5 and NMDA receptor co-activity^27^,^28^. Furthermore, Homer1c expression has shown to reduce mGluR5 dimerisation. We and others have reported altered protein expression of both Homer1b/c and Norbin in the PFC and hippocampus of schizophrenia subjects^23^,^24^,^25^,^29^,^30^. Investigation of Homer1b/c and Norbin expression through neurodevelopment, in particular, in a schizophrenia relevant paradigm, may provide a deeper understanding into potential underlying mechanisms for changes to Group 1 mGluRs localisation and activity.

## Results

**Figure 1:**
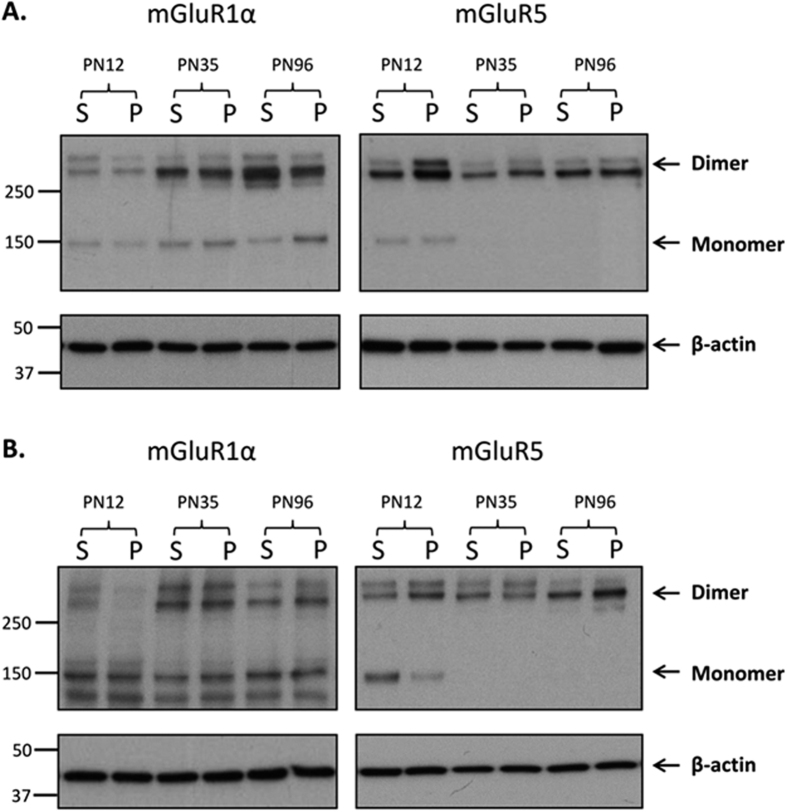
Neurodevelopmental and perinatal PCP-induced effects on Group 1 mGluR protein expression. Representative immunoblot images of mGluR1α and mGluR5 from saline (S) and perinatal phencyclidine (P) treated rats at postnatal days (PN) 12, 35 and 96 in the (**A**) prefrontal cortex and (**B**) hippocampus. NB: Membranes were exposed to film for varying times to optimise images for dimer and monomer quantification. Immunoblot values were normalised to β-actin. Immunoblots of mGluR1α and mGluR5 produced two bands at 270 and 280 kDa, which has been previously reported^33^,^34^ and these were quantified together as the dimer. The band corresponding to 150 kDa was quantified as the monomeric form of mGluR1α and mGluR5, respectively. NB: Immunoblots of mGluR1α in the hippocampus showed a band at approximately 140kda, which was not observed in the PFC. This band was deemed as non-specific and therefore not quantified.

Group 1 mGluRs (dimeric and monomeric forms) were examined at postnatal days (PN)12, 35 and 96, corresponding to juvenile, adolescent and adult time points, which are key periods for the emergence of psychiatric disorders^15^,^31^. mGluR1α and mGluR5 monomeric and dimeric proteins were clearly identified in the rat PFC and hippocampus at all time points (Fig. 1A,B, respectively), with the exception of the mGluR5 monomer, which showed relatively low expression at PN35 and PN96 in both regions. mGluR1α and mGluR5 presented as two dimer bands (~270–280 kDa) and a single monomer band (~150 kDa), as previously reported^9^,^32^,^33^. The two dimeric bands were quantified as the dimer; these bands have previously been reported using alternative mGluR1α and mGluR5 antibodies, and have been confirmed as specific, using respective knockout mice^33^,^34^. The sums of the dimer and monomer densitometry values were taken to equal total mGluR1α and mGluR5.

### mGluR1α total, dimeric and monomeric expression in the PFC and hippocampus

**Figure 2:**
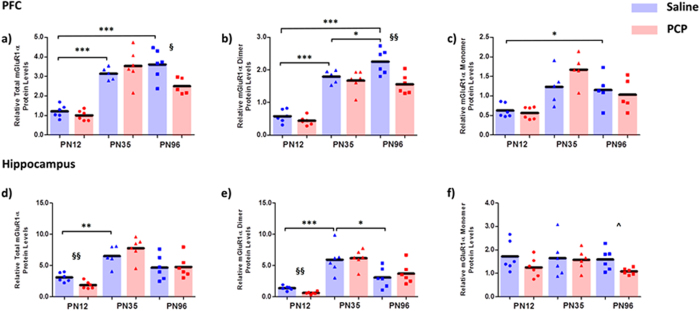
mGluR1α protein expression is developmentally regulated in the PFC and hippocampus and is reduced following perinatal PCP treatment. Relative total, dimeric and monomeric mGluR1α protein levels in the PFC (**a–c**) and hippocampus (**d–f**) of phencyclidine (PCP) treated rats compared to saline controls at postnatal days (PN) 12, 35 and 96 (n = 5–6 per treatment/time point). Bars represent mean values. *p < 0.05, **p < 0.01 and ***p < 0.001 indicate statistical significance between saline treated age groups. ^§^p < 0.05 and ^§§^p < 0.01 indicate statistical significance between perinatal PCP and saline treatment at specific age group. Within the respective age groups the saline group is on the left (blue) and PCP treated group on the right (red).

Two-way ANOVAs revealed significant main effects of age on mGluR1α protein expression in the PFC (Total: F_2,28_ = 55.086; p < 0.001; Dimer: F_2,29_ = 93.889; p < 0.001; Monomer: F_2,26_ = 12.104; p < 0.001). Post-hoc analyses in control rats revealed total mGluR1α expression significantly increased from PN12 to PN35 (160%, p < 0.001) with no significant difference between PN35 and PN96. Dimeric mGluR1α expression similarly increased from PN12 to PN35 in control rats (209%, p < 0.001) followed by a modest increase from PN35 to PN96 (26%, p = 0.049). mGluR1α monomer levels showed a general trend toward increased expression at each developmental time point in the control group, yet a significant difference was only observed between PN12 and PN96 (117%, p = 0.044) (Fig. 2). There was a main effect of PCP treatment on dimeric mGluR1α (F_1,29_ = 12.107; p = 0.002) and age x treatment interactions on total (F_2,28_ = 5.066; p = 0.013) and dimeric mGluR1α protein expression in the PFC (F_2,29_ = 4.244; p = 0.024); PCP treated rodents displayed a reduction in total (−31%, p = 0.018) and dimeric (−31%, p = 0.006) mGluR1α expression compared to saline treated rats at the PN96 time point only (Fig. 2). There was no effect of PCP treatment on mGluR1 monomer.

In the hippocampus mGluR1α total and dimer expression, but not monomer, were significantly affected by age (Total: F_2,29_ = 26.946, p < 0.001; Dimer: F = _2,30_ = 36.222, p < 0.001; Monomer: F_2,30_ = 0.800, p = 0.459). Post-hoc analysis showed mGluR1α protein levels significantly increased between PN12 and PN35 in control rats (Total: 109%, p = 0.007; Dimer: 328%, p < 0.001). This was followed by a decrease from PN35-PN96, which only reached significance for the dimer (Total: −38%, p = 0.095; Dimer: −48% p = 0.024; Fig. 2). There was no main effect of PCP treatment on mGluR1α protein expression in the hippocampus (Total: F_1,29_ = 0.006; p = 0.939; Dimer: F_1,30_ = 0.003; p = 0.959), although there was a trend towards a significant treatment effect on mGluR1α monomer levels (F_1,30_ = 3.870; p = 0.058), in which PCP treatment resulted in an overall 21% reduction in mGluR1α monomer expression. There were no age x treatment interactions for mGluR1α total (F_2,29_ = 1.946; p = 0.161), dimer (F_2,30_ = 0.733; p = 0.489) or monomer (F_2,30_ = 0.635;p = 0.537) protein levels. However, visual inspection of the means suggested treatment effects on mGluR1α protein expression at the PN12 and PN96 time points (Fig. 2). Exploratory post-hoc analyses at the PN12 time point revealed total and dimeric mGluR1α levels were significantly reduced in perinatal PCP treated rats at PN12 (−40%, t_10_ = 3.240; p = 0.009 and −56%, t_10_ = 4.509 p = 0.001, respectively), however mGluR1α monomer levels did not reach significance (t_10_ = 1.470; p = 0.172). Furthermore, monomeric mGluR1α levels were reduced at PN96 in perinatal PCP treated rats (−32%, t_10_ = 2.560; p = 0.028) compared to controls, however this finding did not withstand Bonferroni correction for multiple testing.

### mGluR5 total, dimeric and monomeric expression in the PFC and hippocampus

**Figure 3:**
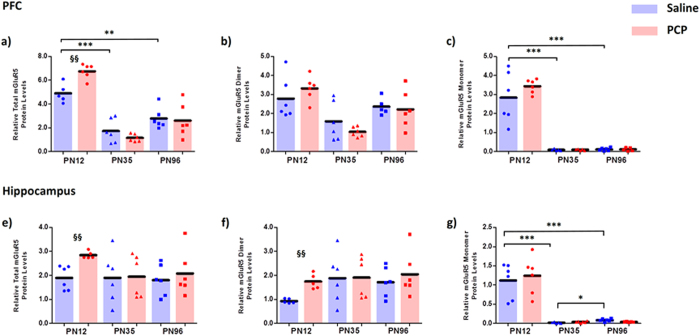
mGluR5 protein expression reduces throughout development and is acutely increased following perinatal PCP treatment in the PFC and hippocampus. Relative total, dimeric and monomeric protein levels mGluR5 in the PFC (**a–c**) and hippocampus (**d–f**) of phencyclidine (PCP) treated rats compared to controls at postnatal days (PN) 12, 35 and 96 (n = 5–6 per treatment/time point). Bars represent mean values. *p < 0.05, **p < 0.01 and ***p < 0.001 indicate statistical significance between saline treated age groups. ^§^p < 0.05 and ^§§^p < 0.01 indicate statistical significance between perinatal PCP and saline treatment at specific age group. Bars represent mean values + SEM. Within the respective age groups the saline group is on the left (blue) and PCP treated group on the right (red).

mGluR5 protein expression in the PFC was strongly influenced by age (Total: F_2,29_ = 71.097, p < 0.001; dimer: F_2,29_ = 13.701, p < 0.001). Total mGluR5 protein expression in the PFC peaked at PN12 in control rats, significantly declining at PN35 (−64%, p < 0.001) with no significant difference between PN35 and PN96 (p = 0.189). While changes in mGluR5 dimer expression in control rats between the developmental stages did not reach statistical significance (F_2,16_ = 2.549; p = 0.114), post-hoc analyses combining the PCP and saline groups showed a significant reduction in mGluR5 dimer from PN12-PN35 (p < 0.001) followed by an increase from PN35-96 (p = 0.021) back to PN12 levels. mGluR5 monomer expression in the PFC showed unequal variances between the groups (F_5,27_ = 20.022, p < 0.001) thus was analysed using non-parametric analyses. There was a significant age effect in control rats (X^2^(2) = 11.014, p = 0.004); peak mGluR5 monomer levels occurred at PN12, followed by a dramatic reduction at PN35 (−97%, U = 0.000, p < 0.001) with levels remaining low at PN96 (Fig. 3). While there were no main effects of PCP treatment on mGluR5 total (F_1,29_ = 1.506, p = 0.230) or dimer (F_1,29_ = 0.035, p = 0.854), total mGluR5 expression in the PFC was influenced by an age x treatment interaction (F_2,29_ = 5.863; p = 0.007) in which perinatal PCP treatment increased total mGluR5 protein expression at PN12 compared to controls (37%, p = 0.002; Fig. 3). This interaction was not observed for the mGluR5 dimer (F_2,29_ = 1.369; p = 0.270). Mann-Whitney testing revealed that perinatal PCP treatment did not significantly influence mGluR5 monomer expression at any of the time points investigated (PN12: U = 14.000, p = 0.522; PN36: U = 11.000, p = 0.754; PN96: U = 14.000, p = 0.931; Fig. 3).

mGluR5 protein expression in the hippocampus showed unequal variance (Total: F_5,28_ = 2.940, p = 0.029; Dimer: F_5,29_ = 3.647, p = 0.011; Monomer: F_5,28_ = 10.273, p < 0.001), therefore non-parametric analyses were employed. There were no significant effects of age on total (X^2^(2) = 2.020, p = 0.364) or dimeric (X^2^(2) = 4.833, p = 0.089) mGluR5 expression. However, similar to the PFC, monomeric mGluR5 expression, showed a strong effect of age (X^2^(2) = 24.939, p < 0.001; Fig. 3). Post-hoc analysis revealed that monomeric mGluR5 expression peaked at PN12 in control rats followed by a significant decline at PN35 (−99%, U = 0.000, p < 0.001) and a modest increase from PN35 to PN96 (+441, U = 1.000, p = 0.011). Mann-Whitney tests revealed that perinatal PCP treatment significantly increased total (50%, U = 0.000, p = 0.006) and dimeric (87%, U = 0.000, p = 0.009), but not monomeric (U = 16.000, p = 0.818) mGluR5 expression at the PN12 time point (Fig. 3). No treatment effect on mGluR5 expression (total, dimer or monomer) was observed at PN35 or PN96 (5 < U < 16; 0.082 < p < 0.0818).

### Expression of Group I mGluR regulators, Homer1b/c and Norbin, in the PFC and hippocampus

**Figure 4:**
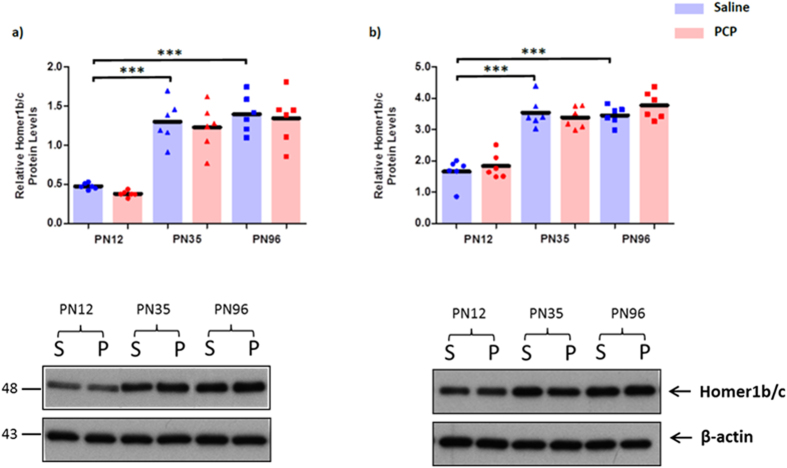
Homer1b/c protein expression is unchanged following perinatal PCP treatment, but is developmentally regulated. Relative protein levels of Homer1b/c in the (**a**) prefrontal cortex and (**b**) hippocampus of phencyclidine (PCP) treated rats compared to controls at postnatal days (PN) 12, 35 and 96 (n = 5–6 per treatment/time point). Bars represent mean values. ***p < 0.001 indicate statistical significance between saline treated age groups. Representative immunoblot images of Homer1b/c from saline (S) and perinatal phencyclidine (P) treated rats at postnatal days (PN) 12, 35 and 96 in the prefrontal cortex (left) and hippocampus (right) are shown below the graphs. Immunoblot values were normalised to β-actin. Homer1b/c immunoblots produced a single band corresponding to its 48 kDa molecular weight. Within the respective age groups the saline group is on the left (blue) and PCP treated group on the right (red).

Homer1b/c levels in the PFC and hippocampus were significantly affected by age (PFC: F_2,30_ = 58.466; p < 0.001; hippocampus: F_2,30_ = 80.713; p < 0.001). In both regions there was a significant increase in Homer1b/c levels between PN12 and PN35 (PFC: 172%; p < 0.001; hippocampus: 113%; p < 0.001) and between PN12 and PN96 (PFC: 192%; p < 0.001; hippocampus: 108%; p < 0.001) (Fig. 4). There were no significant effects of PCP treatment on Homer1b/c levels (PFC: F_1,30_ = 0.909; p = 0.348; hippocampus: F_1,30_ = 0.744; p = 0.395) and no age x treatment interactions (PFC: F_2,30_ = 0.025; p = 0.975; hippocampus: F_2,30_ = 1.095; p = 0.347).

**Figure 5:**
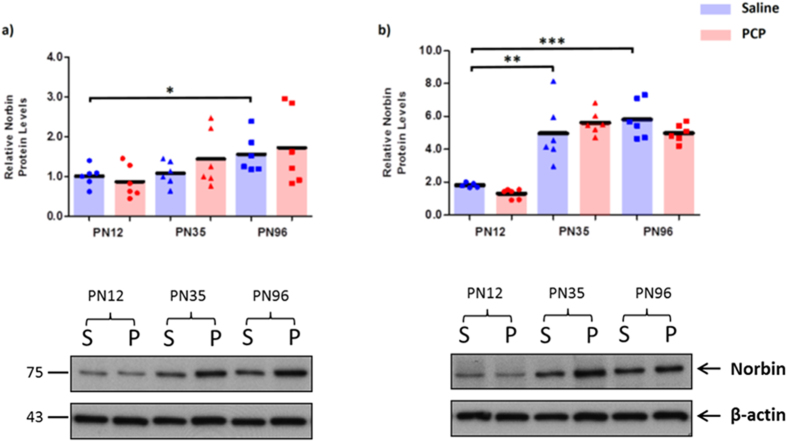
Norbin protein expression is unchanged following perinatal PCP treatment, but is developmentally regulated. Relative protein levels of Norbin in the (**a**) prefrontal cortex and (**b**) hippocampus of phencyclidine (PCP) treated rats compared to controls at postnatal days (PN) 12, 35 and 96 (n = 5–6 per treatment/time point). Bars represent mean values. ***p < 0.001 indicate statistical significance between saline treated age groups. C and D show **r**epresentative immunoblot images of Norbin from saline (S) and perinatal phencyclidine (P) treated rats at postnatal days (PN) 12, 35 and 96 in the prefrontal cortex (left) and hippocampus (right) are shown below the graphs. Immunoblot values were normalised to β-actin. NB: Norbin immunoblots produced a single band corresponding to its 75 kDa molecular weight. Within the respective age groups the saline group is on the left (blue) and PCP treated group on the right (red).

Norbin levels in the PFC and hippocampus were also significantly affected by age (PFC: F_2,30_ = 4.506; p = 0.019; hippocampus: F_2,29_ = 55.676; p < 0.001), with Norbin levels increasing from PN12 to PN96 in the PFC (54%; p = 0.045) and between PN12 and PN36 (173%; p = 0.003) and PN12 to PN96 (219%; p < 0.001) in the hippocampus (Fig. 5). There were no effects of PCP treatment on Norbin expression (PFC: F_1,30_ = 0.477; p = 0.509; hippocampus: F_1,29_ = 0.468; p = 0.499) and no age x treatment interactions (PFC: F_2,30_ = 0.588;p = 0.562; hippocampus: F_2,29_ = 1.892; p = 0.169).

## Discussion

In this study, we report the neurodevelopmental profile of Group 1 mGluRs, including dimeric and monomeric forms, in the PFC and hippocampus. Furthermore, we detailed the effects of perinatal PCP treatment, an established neurodevelopmental model of schizophrenia, on Group 1 mGluR protein expression in these brain regions. Whilst mGluR1α and mGluR5 share similar structural and functional homology^35^, we report here that they display differential monomeric and dimeric neurodevelopmental profiles and expression patterns following perinatal PCP treatment in rats. In view of the recent literature regarding mGluR-targeting compounds for the treatment of various psychiatric and neurodevelopmental disorders^36^, the present results may have implications for the pharmacological profiles of these compounds at specific ages.

During development, total mGluR1α protein and importantly its functional dimeric form, is relatively low in the PFC and hippocampus following birth, with expression peaking at adolescence (hippocampus) or adulthood (PFC). This is largely consistent with the one previous study examining dimeric and monomeric mGluR1α in cortex and hippocampus^10^. mGluR5 dimer levels were relatively abundant and stable throughout the three stages of development examined. Whilst mGluR5 monomers were expressed at the juvenile time point, they dramatically reduced at adolescence and adulthood, to the lowest limits of detection in both the hippocampus and PFC. Further investigation is required to determine if this trend is specific to certain cell types. However, we also report that this abundance of monomeric mGluR5 early in neurodevelopment is also present in the nucleus accumbens region (see Supplementary Fig. S1), which is characterised by a vastly different neuronal population and neuronal network than the PFC^37^ and hippocampus^38^ suggesting this finding is not brain region specific.

Previous studies have reported a more subtle reduction over time when examining total mGluR5 expression in these regions^7^,^10^,^39^, however this is the first time the expression of the mGluR5 monomer has been reported under non-reduced conditions, relative to the dimeric form during neurodevelopment. These dimeric and monomeric neurodevelopmental expression profiles may have implications for the mGluR pharmacological profiles at specific ages. Positive allosteric modulators (PAMs), particularly targeting mGluR5, are being developed for the potential treatment of schizophrenia^36^. mGluR5 PAMs bind within the 7-transmembrane membrane region of mGluR5 and whilst exerting no G-protein activation alone when bound to dimers, PAMs are capable of potentiating glutamatergic signalling when glutamate is bound. In contrast, recent works from the Pin research team have demonstrated that monomeric mGluRs are capable of G-protein activity, similar to that of a full agonist response, in the presence of PAMs and the absence of glutamate^12^. This is important since glutamate-induced neurotoxic effects and seizures have been observed following the use of Group 1 mGluR agonists^40^,^41^,^42^. The absence of the monomer at adolescent and adult time points confers a potential advantage in avoiding these agonist-like effects of mGluR5 PAMs. It is not known if the same abundance of monomeric mGluR5 is present in humans or at what neurodevelopmental time point monomeric expression is reduced, however administration of mGluR5 PAMs at a time point when monomeric mGluR5 is highly expressed may have adverse consequences. Further studies will need to explore whether the abundant monomeric expression is capable of reaching the cell surface where it is accessible to endogenous and exogenous targets. We investigated the Group I mGluR regulators Homer1b/c and Norbin, which can regulate trafficking of mGluR5 to the cell surface^26^. Interestingly, these endogenous regulators were in lower abundance at PN12 in both the PFC and hippocampus, at a time when there was high monomer, suggesting possible differences in regulation and trafficking of mGluR5 at the different developmental stages.

Perinatal PCP treatment substantially increased total mGluR5 expression in the PFC and hippocampus at the juvenile time period, yet this did not extend to adolescence or adulthood time points. These findings are consistent with postnatal administration of the NMDA receptor antagonist, MK-801, which increased mGluR5 mRNA expression in the cortex and hippocampus, 4 hrs post treatment^43^. Whilst the aforementioned study did not investigate the long-term effect on mGluR5 mRNA or protein levels, Owaczarek *et al*., reported that perinatal PCP treatment had no long term effect on mGluR5 protein levels in the PFC or hippocampus, when measured at the single adulthood time point, consistent with our findings^17^. Together, these results suggest that perinatal NMDA receptor antagonism may acutely upregulate cortical and hippocampal mGluR5 mRNA and protein expression, but does not affect its long-term expression at adulthood. Despite inducing acute alterations in mGluR5 expression, changes at such a critical neurodevelopmental period may have long-term consequences on excitatory/inhibitory tone. Investigation of mGluR5 KO mice has shown mGluR5 activation critical for driving the normal neurodevelopmental NMDA receptor subunit, NR2B/2A switch^44^. The composition of NR2A/B subunits plays an important role in the calcium dynamics of the NMDA receptor and synaptic plasticity^45^,^46^. Although, we observed an increase in mGluR5 expression following perinatal PCP treatment, it is possible that alterations in mGluR5 expression and/or activity during the juvenile neurodevelopmental period may consequently alter the development of NMDA receptor subunit composition, which has indeed been extensively reported in this model^14^,^15^,^17^.

In contrast to mGluR5, the present study reported that total mGluR1α expression was reduced in the juvenile hippocampus, of perinatal PCP treated rats. mGluR1α and mGluR5 share similar sequence and second messenger coupling homology, although several studies have identified differences in their cellular location, intracellular signalling and functionality^47^,^48^,^49^. In addition, we have shown in the present study that they have differential dimeric and monomeric neurodevelopmental profiles, suggesting that the regulation of dimerization may differ. Similar to mGluR5, mGluR1α is co-localised with the NMDA receptor and can modulate its activity^50^. However, in contrast to mGluR5, which is abundantly found throughout the hippocampus and on various cell types, mGluR1α mRNA and protein expression is found primarily in the CA1 region and localises to GABAergic interneurons^49^,^51^,^52^,^53^. Hippocampal interneurons have previously been reported to be prone to insult and neuronal death, following perinatal PCP treatment^54^ and reductions in hippocampal mGluR1α may be a consequence of a reduction in interneuron cell number. The results from the present study largely suggest that perinatal PCP treatment causes acute changes in mGluR1α and mGluR5 at the early stages of perinatal neurodevelopment, which may contribute to altered glutamatergic signalling and neurodevelopmental abnormalities previously reported in this model^14^,^15^,^17^.

In summary, we report that Group 1 mGluRs, mGluR1α and mGluR5, exhibit differential temporal and regional neurodevelopmental expression patterns, especially in relation to dimeric and monomeric expression. These findings may have implications for the effectiveness and side effect profile of mGluR PAMs at specific developmental stages, however, pharmacological studies are required to investigate this. Furthermore, we show that perinatal PCP treatment disrupts the normal developmental expression of these proteins in the PFC and hippocampus, particularly at the juvenile period; this may contribute to ongoing developmental glutamatergic alterations that are believed to contribute to the development of schizophrenia.

## Materials and Methods

### Animals

Pregnant Sprague-Dawley rats were obtained at gestational day 14 from the Animal Resources Centre (WA, Australia). Animals were housed under constant temperature control (20 °C) and 12:12 hour light-dark cycle. Rats were provided food and water *ad libitum*. Pups were sexed on post-natal day (PN) 7 and subsequently allocated into PCP or saline treatment groups. Dam and littermates were housed together until 3 weeks of age, when they were weaned and housed 2/per cage of the same sex and treatment. Only female rats were used in subsequent protein analyses in this study. This study was carried out in accordance with the Australian Code of Practice for the Care and Use of Animals for Scientific Purposes (8th edition) and was approved by the University of Wollongong Animal Ethics Committee (AE13/03).

### Perinatal PCP Treatment

Female Sprague-Dawley rats were administered PCP (10 mg/kg, s.c.) (Sigma, Castle Hill, Australia) or saline (0.9% NaCl) on PN7, 9 and 11. Rats were subsequently euthanised by carbon dioxide asphyxiation and decapitation on PN12, 35 and 96 (n = 6/group) coinciding with juvenile, adolescent and adult time points as previously described in Du Bois *et al*.,^15^,^31^. The prefrontal cortex and hippocampus were dissected according to a standard rat brain atlas^55^, snap frozen in liquid nitrogen and stored at −80 °C.

### Rat Brain Tissue Preparation

Tissue samples (left and right hemispheres combined) were homogenised in buffer containing 0.1 M Tris-HCl, 2 mM EDTA, 10% glycerol, 2% SDS, 100 mM iodoacetamide, 0.5 mM PMSF, Protease Inhibitor Cocktail (P8340; Sigma, Australia) and Phosphatase Inhibitor Cocktail 2 (Sigma, Australia). Samples were then centrifuged at 10 000 g for 10 minutes at 4 °C, supernatant collected and stored at −80 °C. Total protein concentration was determined using a DC assay kit as per the manufacturer’s instructions (Bio-Rad, Australia).

### Immunoblotting

Equal amounts of protein (2.5 ug/well) were separated in 4–20% TGX precast gels (Bio-Rad, Australia) under non-reducing conditions to promote the integrity of mGluR homodimers. Proteins were subsequently transferred onto PDVF membranes (Bio-Rad) and membranes blocked with 5% BSA or skim milk (w/v) for 60 minutes at room temperature. The membranes were incubated overnight in the primary polyclonal antibodies at the following concentrations: anti-mGluR1α (1:15000; D5H10, Cell Signalling), anti-mGluR5 (1:5000; ab29170, Abcam), anti-Neurochondrin (1:7500; ab130507, Abcam) and anti-Homer1b/c (1:7500; ab211415, Abcam). Membranes were subsequently incubated with horseradish peroxidase conjugated secondary antibodies (1:5000; AP307P, Millipore). Bands were visualised using Amersham ECL western blotting detection reagent (GE Healthcare, Australia) and membranes exposed to Hyperfilm (GE Healthcare, Australia). Exposure times were reliant on obtaining optimal images to quantify Group 1 mGluR dimeric and monomeric bands. Films were scanned using a GS-800 scanner (Bio-Rad) and densitometry values were quantified. Relative densitometry values for each protein were normalised to their respective β-actin levels and an internal control value, to account for protein loading and gel-gel variability, respectively. Each sample was run in duplicate.

### Statistical Analysis

Two-way Analyses of Variance (ANOVA) were used to determine any effects of age and treatment on relative protein densities. Where significant interactions were found, independent t-tests were used to identify differences between treatment groups at specific ages. One-way ANOVAs were used to determine significance between age groups within the control group only. While all data showed a normal distribution (Kolmogorov–Smirnov p > 0.05), normality testing can be unreliable in small samples^56^. However, given the findings from the KS test coupled with the observation that biological variables tend to be normally distributed^57^, statistical analyses were completed using parametric analyses as per Dean *et al*.^58^ and our previous studies^15^. However, where there were unequal variances between the groups (mGluR5 monomer in the PFC, mGluR5 total, dimer and monomer in the hippocampus), non-parametric analyses were employed. Kruskall-Wallis tests were used to determine the significance between age groups within the control groups. Mann-Whitney U tests were used to determine the effect of PCP treatment at specific ages. Significance was set at an alpha level of *p = 0.05*.

## Additional Information

**How to cite this article**: Lum, J. S. *et al*. Neurodevelopmental Expression Profile of Dimeric and Monomeric Group 1 mGluRs: Relevance to Schizophrenia Pathogenesis and Treatment. *Sci. Rep.* **6**, 34391; doi: 10.1038/srep34391 (2016).

## Supplementary Material

Supplementary Information
