# Supplementary material for: Neurodevelopmental Expression Profile of Dimeric and Monomeric Group 1 mGluRs: Relevance to Schizophrenia Pathogenesis and Treatment
Source: Sci Rep. 2016 Oct 10;6:34391. doi: 10.1038/srep34391 (PMC5056358; doi:10.1038/srep34391)
Supplement: Supplementary Information [file srep34391-s1.pdf]

Neurodevelopmental Expression Profile of Dimeric and Monomeric Group 1 mGluRs: Relevance to Schizophrenia Pathogenesis and Treatment

Jeremy S. Lum[a,d], Francesca Fernandez[a,c,d] , Natalie Matosin[a,d,e], Jessica L. Andrews[a,d], Xu-Feng Huang[a,d], Lezanne Ooi[a,b]and Kelly A. Newell[a,d*]

mGluR5 Neurodevelopment in the Nucleus Accumbens

The neurodevelopmental profile of total mGluR5 levels showed a significant age effect (X2=11.920, p=0.003), as did monomeric mGluR5 levels (X2=11.705, p=0.003), however dimeric mGluR5 showed no age effect (X2=0.574, p=0.751). Total mGluR5 expression peaked at PN12, whereby its expression significantly reduced at PN35 (p=0.004), where levels remained stable at PN96. Similar to all other regions examined, mGluR5 monomeric levels peaked at PN12 and drastically reduced at PN35 (p=0.003).

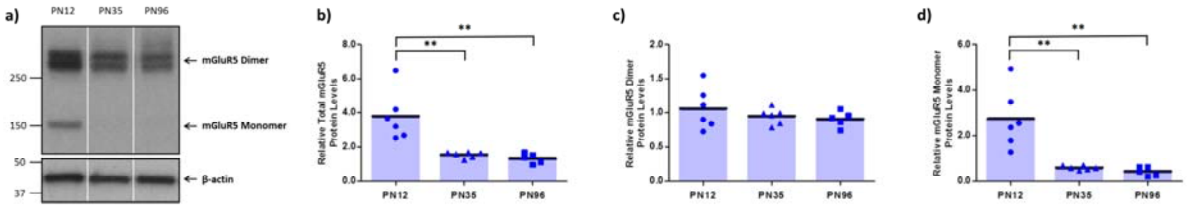

Supplementary Figure S1: Monomeric mGluR5 protein levels in the nucleus accumbens follows a similar neurodevelopmental trend as the prefrontal cortex and hippocampus. a) Representative immunoblot images of mGluR1α and mGluR5 from saline (S) treated rats at postnatal days (PN) 12, 35 and 96 in the nucleus accumbens. Immunoblots of mGluR1α and mGluR5 produced two bands at 270 and 280 kDa, these were quantified together as the dimer. The band corresponding to 150 kDa was quantified as the mGluR5 monomer. Relative neurodevelopmental protein levels of **a)** total, **b)** dimeric and **c)** monomeric mGluR5 in the nucleus accumbens at postnatal days (PN) 12, 35 and 96 (n=5-6 per time point). Bars represent mean values. **p<0.01.
